# Charlson comorbidity Index for descriptive risk stratification of long-term All-cause mortality in elderly patients with acute myocardial infarction: a retrospective cohort study

**DOI:** 10.3389/fcvm.2026.1852843

**Published:** 2026-07-17

**Authors:** Fengxiang Han, Pengyuan Zhao, Yukun Xia, Xiaoxu Liu, Liting Yin, Shuxia Liu, Sheng Guo

**Affiliations:** 1 Department of Geriatrics, Sinopharm Tongmei General Hospital, Datong, Shanxi, China; 2 Department of Clinical Laboratory, Sinopharm Tongmei General Hospital, Datong, Shanxi, China

**Keywords:** acute myocardial infarction, all-cause mortality, charlson comorbidity index, comorbidity burden, descriptive risk stratification, elderly

## Abstract

**Background:**

Elderly patients with acute myocardial infarction (AMI) commonly present with multiple coexisting chronic conditions. The Charlson Comorbidity Index (CCI) is a well-validated tool for quantifying comorbidity burden; however, its role in descriptive long-term risk stratification among elderly patients with AMI remains insufficiently characterized.

**Methods:**

We conducted a retrospective cohort study of 350 elderly patients (age ≥65 years) admitted with AMI to Sinopharm Tongmei General Hospital between January 2020 and December 2023. CCI scores were calculated from admission records, and patients were stratified into low (CCI 1–2, *n* = 118), moderate (CCI 3–4, *n* = 142), and high (CCI ≥5, *n* = 90) comorbidity groups. The primary endpoint was all-cause mortality during follow-up. Cox proportional hazards models were used to describe the association between CCI-defined comorbidity burden and long-term all-cause mortality. Two separate multivariable models were constructed: Model 1 incorporated the CCI total score with non-CCI covariates, and Model 2 replaced the CCI total score with selected individual comorbidity components to avoid collinearity.

**Results:**

Over a median follow-up of 40.9 months (range: 1.4–57.5 months), 98 patients (28.0%) died. All-cause mortality increased stepwise across CCI groups: 16.95% in the low, 29.58% in the moderate, and 40.00% in the high comorbidity group (Log-rank *P* < 0.001). On univariable Cox analysis, CCI score was significantly associated with all-cause mortality (HR = 1.164, 95% CI 1.059–1.278, *P* = 0.002). In multivariable Model 1 (CCI + non-CCI covariates), smoking history (HR = 1.862, *P* = 0.003) and PCI receipt (HR = 0.497, *P* = 0.001) were independently associated with long-term all-cause mortality; however, CCI did not reach statistical significance after adjustment for age and other confounders (HR = 1.089, *P* = 0.125). Exploratory subgroup analyses suggested that patients with comorbid chronic kidney disease (CKD) or heart failure had higher observed mortality risk, but these subgroup findings were not intended to establish independent predictive ability.

**Conclusions:**

In this retrospective cohort of elderly patients with AMI, higher CCI scores were associated with greater long-term all-cause mortality and adverse clinical outcomes in univariable and Kaplan–Meier analyses, supporting the use of CCI as a simple descriptive risk-stratification tool for identifying patients with high comorbidity burden. However, CCI was not independently associated with long-term mortality after multivariable adjustment; therefore, these findings should not be interpreted as confirmatory evidence that CCI independently predicts long-term mortality. Smoking history was independently associated with worse prognosis, whereas PCI receipt was associated with lower observed mortality after adjustment; the latter association should be interpreted cautiously because of potential indication bias, contraindications, treatment-selection effects, and residual confounding inherent to the observational study design. Routine CCI assessment may assist in characterizing comorbidity burden, but individualized risk assessment should integrate age, AMI severity, renal function, LVEF, PCI eligibility, treatment preferences, and other relevant clinical factors.

## 1 Introduction

Acute myocardial infarction (AMI) is a leading cause of mortality and cardiac dysfunction in older adults, and its incidence continues to rise with global population aging (1). Compared with younger patients, elderly individuals with AMI are disproportionately affected by multiple concurrent chronic diseases—a state termed multimorbidity—including hypertension, diabetes mellitus, chronic kidney disease (CKD), and heart failure (2, 3). This comorbidity burden substantially complicates clinical decision-making, increases the risk of adverse drug interactions, and is associated with markedly higher peri-procedural and long-term mortality (4).

The Charlson Comorbidity Index (CCI) is a widely used instrument for quantifying comorbidity burden by assigning weighted scores to predefined conditions and summing them into a composite measure (5, 6). Although CCI has demonstrated prognostic value across various clinical settings—including oncology, critical care, and chronic disease management—its utility in elderly AMI populations remains underexplored. Existing evidence is largely limited to short-term or in-hospital outcomes and lacks robust long-term follow-up data (7).

In this study, we conducted a retrospective cohort study of elderly hospitalized patients with AMI at a tertiary hospital in China to describe the association between CCI-defined comorbidity burden and long-term all-cause mortality. CCI scores were calculated at admission, and patients were stratified into different comorbidity-burden groups according to their CCI scores. We systematically compared baseline clinical characteristics, in-hospital outcomes, long-term all-cause mortality, and major adverse cardiovascular events (MACE) across these groups. Cox proportional hazards regression models were used to examine whether the association between CCI and mortality persisted after adjustment for age and other clinical covariates. In addition, exploratory subgroup analyses were performed to describe the consistency of risk stratification across different clinical strata. The primary aim of this study was descriptive risk stratification rather than development or confirmation of an independent mortality prediction model.

## 2 Methods

### 2.1 Study design and population

This retrospective cohort study was approved by the Ethics Committee of Sinopharm Tongmei General Hospital (approval number: 2023-K-08) and conducted in accordance with the Declaration of Helsinki. Using the hospital's electronic medical record system and cardiovascular disease registry, we identified all patients aged ≥65 years who were admitted to the cardiology department and diagnosed with AMI between January 2020 and December 2023.

Inclusion criteria were: (1) age ≥65 years; (2) AMI diagnosis per the Fourth Universal Definition of Myocardial Infarction (8); (3) dynamic elevation of cardiac troponin on admission; (4) receipt of standardized in-hospital care; and (5) complete follow-up data (for patients who survived, follow-up duration ≥6 months; patients who died within 6 months were included with their actual survival time).

Exclusion criteria were: (1) inter-hospital transfer cases (either direction); (2) hemodynamic instability due to malignant arrhythmia or severe aortic stenosis; (3) in-hospital death within 48 h of admission attributable to multi-organ failure (to minimize early mortality bias); (4) severe psychiatric illness or cognitive impairment precluding follow-up. After applying these criteria, 350 patients were enrolled. All included variables had complete data with no missing values. Follow-up data were collected through outpatient visit records and telephone interviews; the follow-up cutoff date was June 30, 2025.

### 2.2 CCI scoring and comorbidity grouping

CCI scores were calculated based on admission medical records, prior diagnosis history, and available ancillary test results. The following comorbidities were assessed per the standard CCI framework: myocardial infarction, congestive heart failure, peripheral vascular disease, cerebrovascular disease/TIA, dementia, chronic pulmonary disease, connective tissue disease, peptic ulcer disease, mild liver disease, diabetes mellitus without end-organ damage, diabetes with end-organ damage, hemiplegia, moderate-to-severe renal disease/CKD, solid tumor without metastasis, leukemia/lymphoma, moderate-to-severe liver disease, and metastatic solid tumor. Each condition was assigned its designated weight, and the scores were summed to obtain the total CCI. Of note, the index AMI event was included as the myocardial infarction component in the CCI calculation. Because all patients had AMI, this uniformly shifted all CCI scores upward by 1 point without changing the relative ranking of patients according to comorbidity burden. However, this approach may affect the interpretation of absolute CCI values and predefined CCI categories, because CCI is generally intended to reflect pre-existing comorbidity burden rather than the acute index disease itself. Patients were classified into three groups: low comorbidity (CCI 1–2, *n* = 118), moderate comorbidity (CCI 3–4, *n* = 142), and high comorbidity (CCI ≥5, *n* = 90) (9).

### 2.3 Data collection and outcomes

Variables collected included: age, sex, body mass index (BMI), smoking history, AMI type (STEMI vs. NSTEMI), LVEF, peak troponin, serum creatinine, receipt of percutaneous coronary intervention (PCI), and CCI score. The primary endpoint was all-cause mortality during follow-up. Secondary endpoints included major adverse cardiovascular events (MACE), defined as a composite endpoint of cardiovascular death, non-fatal recurrent myocardial infarction, stroke, or heart failure rehospitalization, as well as all-cause readmission.

### 2.4 Statistical analysis

Statistical analyses were performed using SPSS version 26.0. Continuous variables were tested for normality using the Shapiro–Wilk test. Normally distributed variables are presented as mean ± standard deviation and compared across groups using one-way ANOVA; non-normally distributed variables are expressed as median (interquartile range) and compared using the Kruskal–Wallis test. Categorical variables are presented as frequency and percentage and compared using the chi-squared test or Fisher's exact test. The primary survival analysis used Cox proportional hazards models, with follow-up time as the time variable and all-cause death as the endpoint. Patients who were alive at the follow-up cutoff date were censored. Univariable Cox analyses were first performed for all candidate variables. Variables associated with mortality in univariable analyses, together with clinically relevant covariates selected *a priori*, were entered into two separate multivariable Cox models. Model 1 included the CCI total score plus non-CCI covariates, including age, sex, BMI, smoking history, LVEF, PCI receipt, and hypertension, to examine whether the association between the composite CCI and mortality persisted after adjustment for clinical covariates. Model 2 replaced the CCI total score with selected individual comorbidity components, including stroke/TIA and peripheral vascular disease, to avoid collinearity between the composite CCI score and its component conditions. The proportional hazards assumption was assessed using appropriate diagnostic methods, and no substantial violation was observed. Kaplan–Meier survival curves were estimated and compared using log-rank tests. Pre-specified subgroup analyses were designated as exploratory and were used to describe the consistency of the association between CCI score and mortality across clinical strata. These subgroup analyses were not intended as confirmatory evidence of independent predictive ability. Interaction *P* values were reported to assess potential effect modification. All included variables had complete data (100% completeness; no imputation required). A two-tailed *P* < 0.05 was considered statistically significant.

## 3 Results

### 3.1 Baseline characteristics

Of 350 elderly AMI patients enrolled, 118 were in the low, 142 in the moderate, and 90 in the high comorbidity group. Overall, 204 patients (58.3%) were male. Groups were well balanced with respect to sex distribution, BMI, and smoking history (all *P* > 0.05). However, statistically significant differences in age, LVEF, and peak troponin across CCI strata indicate residual clinical heterogeneity; subsequent results should therefore be interpreted cautiously in the context of multivariable adjustment. Mean age increased progressively across groups (*P* < 0.001). LVEF declined significantly with higher CCI scores, while peak troponin and CCI scores increased accordingly (all *P* < 0.001). There was no significant difference in PCI rates across groups (*P* = 0.120). Baseline characteristics are summarized in Table 1.

**Table 1:** Baseline characteristics by comorbidity group.

Variable	Low (*n* = 118)	Moderate (*n* = 142)	High (*n* = 90)	F/*χ*^2^	*P* value
Age (years)	70.58 ± 5.52	72.92 ± 6.17	75.63 ± 6.96	11.834	<0.001
Male, *n* (%)	72 (61.0)	83 (58.5)	49 (54.4)	1.243	0.537
BMI (kg/m^2^)	24.12 ± 3.19	24.38 ± 3.25	23.85 ± 3.48	0.714	0.490
Smoking history, *n* (%)	54 (45.8)	67 (47.2)	41 (45.6)	0.115	0.944
STEMI, *n* (%)	73 (61.9)	84 (59.2)	56 (62.2)	0.298	0.862
LVEF (%)	54.28 ± 8.50	51.63 ± 9.15	47.38 ± 10.30	16.542	<0.001
Peak troponin (ng/mL)	12.87 ± 8.36	15.55 ± 10.12	18.84 ± 12.35	9.218	<0.001
PCI performed, *n* (%)	86 (72.9)	96 (67.6)	54 (60.0)	4.237	0.120
CCI score	1.66 ± 0.48	3.43 ± 0.50	6.23 ± 1.20	892.341	<0.001

CCI, charlson comorbidity index; STEMI, ST-elevation myocardial infarction; LVEF, left ventricular ejection fraction; PCI, percutaneous coronary intervention; BMI, body mass index. STEMI, ST-segment elevation myocardial infarction.

### 3.2 Distribution of comorbidities

The prevalence of all major comorbidities increased progressively with higher CCI scores (all *P* < 0.001). CKD was present in 10.2%, 30.3%, and 60.0% of patients in the low, moderate, and high comorbidity groups, respectively. The corresponding rates for heart failure were 6.8%, 25.4%, and 57.8%, and for malignancy were 5.1%, 12.7%, and 26.7%. Full comorbidity distribution data are presented in Table 2.

**Table 2:** Prevalence of major comorbidities across groups, *n* (%).

Comorbidity	Low (*n* = 118)	Moderate (*n* = 142)	High (*n* = 90)	χ^2^	*P* value
Hypertension	72 (61.0)	104 (73.2)	76 (84.4)	13.427	0.001
Diabetes mellitus	38 (32.2)	67 (47.2)	58 (64.4)	20.348	<0.001
Chronic kidney disease	12 (10.2)	43 (30.3)	54 (60.0)	63.219	<0.001
Heart failure	8 (6.8)	36 (25.4)	52 (57.8)	76.841	<0.001
Stroke/TIA	14 (11.9)	38 (26.8)	42 (46.7)	33.712	<0.001
COPD	16 (13.6)	34 (23.9)	38 (42.2)	23.184	<0.001
Peripheral vascular disease	10 (8.5)	28 (19.7)	36 (40.0)	30.427	<0.001
Malignancy	6 (5.1)	18 (12.7)	24 (26.7)	22.371	<0.001

TIA, transient ischemic attack; COPD, chronic obstructive pulmonary disease. *P* values calculated by chi-squared or Fisher's exact test.

### 3.3 In-Hospital outcomes

Length of hospital stay increased with CCI category (*P* < 0.001). In-hospital mortality, acute heart failure, cardiogenic shock, acute kidney injury, and in-hospital MACE all increased significantly with comorbidity burden (all *P* < 0.05). The in-hospital MACE rate reached 53.3% in the high comorbidity group vs. 11.9% in the low comorbidity group (*P* < 0.001). Given the observational retrospective design, these between-group differences should be interpreted as associations between comorbidity burden and clinical outcomes rather than as evidence of causation. Admission creatinine levels were also markedly higher in the high comorbidity group, reflecting greater baseline renal impairment (*P* < 0.001). Details are shown in Table 3.

**Table 3:** In-hospital clinical outcomes by comorbidity group.

Outcome	Low (*n* = 118)	Moderate (*n* = 142)	High (*n* = 90)	F/χ^2^	*P* value
Length of stay (days)	7.22 ± 2.16	9.85 ± 3.54	13.41 ± 5.26	64.317	<0.001
In-hospital death, *n* (%)	2 (1.7)	6 (4.2)	10 (11.1)	12.842	0.002
Acute heart failure, *n* (%)	10 (8.5)	28 (19.7)	38 (42.2)	38.241	<0.001
Cardiogenic shock, *n* (%)	4 (3.4)	12 (8.5)	18 (20.0)	19.847	<0.001
Acute kidney injury, *n* (%)	6 (5.1)	22 (15.5)	32 (35.6)	38.714	<0.001
In-hospital MACE, *n* (%)	14 (11.9)	36 (25.4)	48 (53.3)	46.382	<0.001
Creatinine (µmol/L)	84.37 ± 18.32	102.57 ± 28.58	138.74 ± 47.08	58.214	<0.001

MACE, major adverse cardiovascular events, defined as a composite endpoint of cardiovascular death, non-fatal recurrent myocardial infarction, stroke, or heart failure rehospitalization.

### 3.4 Follow-up outcomes

The median follow-up durations were 39.8, 41.3, and 42.0 months in the low, moderate, and high comorbidity groups, respectively, with an overall median follow-up of 40.9 months (range: 1.4–57.5 months). During follow-up, 98 patients (28.0%) died: 20 (16.95%) in the low, 42 (29.58%) in the moderate, and 36 (40.0%) in the high comorbidity group. Kaplan–Meier analysis confirmed a significant stepwise reduction in survival across groups (Log-rank *χ*^2^ = 16.824, *P* < 0.001). Heart failure rehospitalization and total MACE rates also differed significantly across groups (*P* < 0.001). The between-group difference in stroke incidence did not reach statistical significance (*P* = 0.054), which may reflect limited statistical power. Full follow-up outcome data are presented in Table 4.

**Table 4:** Follow-up outcomes by comorbidity group, *n* (%).

Outcome	Low (*n* = 118)	Moderate (*n* = 142)	High (*n* = 90)	Log-rank χ^2^	*P* value
All-cause death	20 (16.95)	42 (29.58)	36 (40.00)	16.824	<0.001
Cardiovascular death	12 (10.2)	26 (18.3)	24 (26.7)	11.347	0.003
Non-fatal recurrent MI	14 (11.9)	24 (16.9)	22 (24.4)	6.218	0.044
Stroke	8 (6.8)	18 (12.7)	16 (17.8)	5.847	0.054
HF rehospitalization	10 (8.5)	32 (22.5)	34 (37.8)	25.641	<0.001
Total MACE	38 (32.2)	74 (52.1)	66 (73.3)	30.472	<0.001
All-cause readmission	42 (35.6)	84 (59.2)	62 (68.9)	22.318	<0.001

MACE, major adverse cardiovascular events, defined as a composite endpoint of cardiovascular death, non-fatal recurrent myocardial infarction, stroke, or heart failure rehospitalization; HF, heart failure; MI, myocardial infarction.

### 3.5 Univariable analysis of risk factors for long-term All-cause mortality

As shown in Table 5, univariable Cox regression analysis identified CCI score (HR = 1.164, 95% CI: 1.059–1.278, *P* = 0.002), age (HR = 1.042, *P* = 0.007), smoking history (HR = 1.653, *P* = 0.014), PCI receipt (HR = 0.484, *P* < 0.001), and peripheral vascular disease (HR = 1.554, *P* = 0.049) as factors significantly associated with long-term all-cause mortality. Male sex, BMI, LVEF, hypertension, and stroke/TIA were not statistically significant in the univariable analysis (all *P* > 0.05). It should be emphasized that these univariable associations represent unadjusted estimates and cannot alone be interpreted as evidence that CCI independently predicts long-term mortality.

**Table 5:** Univariable Cox regression analysis of risk factors for long-term all-cause mortality.

Variable	HR	95% CI	*P* value
CCI score	1.164	1.059～1.278	0.002
Age, years	1.042	1.012～1.074	0.007
Male sex	0.879	0.590～1.312	0.529
BMI, kg/m^2^	0.967	0.910～1.028	0.284
Smoking history	1.653	1.107～2.467	0.014
LVEF, %	0.989	0.969～1.009	0.264
PCI performed	0.484	0.325～0.720	<0.001
Hypertension	1.320	0.821～2.122	0.252
Stroke/TIA	1.151	0.749～1.769	0.521
Peripheral vascular disease	1.554	1.002～2.410	0.049

### 3.6 Multivariable analysis of risk factors for long-term All-cause mortality

Variables associated with mortality in univariable analyses, together with clinically relevant covariates selected *a priori*, were entered into multivariable Cox proportional hazards models. Two separate models were constructed: Model 1 included the CCI total score and non-CCI covariates, whereas Model 2 replaced the CCI total score with selected individual comorbidity components.

In Model 1, smoking history was independently associated with higher long-term all-cause mortality, whereas PCI receipt was associated with lower observed mortality after adjustment. After adjustment for age and other covariates, the association between CCI score and long-term all-cause mortality was attenuated and did not reach statistical significance (HR = 1.089, 95% CI: 0.977–1.214, *P* = 0.125). This finding suggests that the unadjusted association between CCI and mortality may be partly explained by age and other clinical covariates. Age also showed a borderline trend toward significance (HR = 1.033, *P* = 0.056). Therefore, CCI should be interpreted in this study as a descriptive risk-stratification marker rather than as a confirmed independent predictor of long-term mortality. The results of Model 1 are shown in Table 6.

**Table 6:** Multivariable Cox regression analysis of risk factors for long-term all-cause mortality (model 1: CCI total score + non-CCI covariates).

Variable	HR	95% CI	*P* value
CCI score	1.089	0.977～1.214	0.125
Age, years	1.033	0.999～1.067	0.056
Male sex	0.841	0.560～1.262	0.403
BMI, kg/m^2^	0.953	0.896～1.013	0.122
Smoking history	1.862	1.238～2.801	0.003
LVEF, %	1.003	0.982～1.025	0.756
PCI performed	0.497	0.330～0.748	0.001
Hypertension	1.217	0.751～1.973	0.426

Model 1 included CCI score, age, sex, BMI, smoking history, LVEF, PCI receipt, and hypertension. HR indicates hazard ratio. All continuous variables were entered into the model using their original units.

In Model 2, in which individual comorbidities replaced the CCI total score, age (HR = 1.042, 95% CI: 1.010–1.076, *P* = 0.011), smoking history (HR = 1.893, *P* = 0.002), and PCI receipt (HR = 0.471, *P* < 0.001) were independent prognostic factors. Stroke/TIA and peripheral vascular disease did not remain statistically significant after multivariable adjustment (*P* > 0.05). The results of Model 2 are shown in Table 7.

**Table 7:** Multivariable Cox regression analysis of risk factors for long-term all-cause mortality (model 2: individual comorbidities replacing the CCI total score).

Variable	HR	95% CI	*P* value
Age, years	1.042	1.010～1.076	0.011
Male sex	0.831	0.554～1.248	0.372
BMI, kg/m^2^	0.947	0.892～1.006	0.078
Smoking history	1.893	1.257～2.853	0.002
LVEF, %	1.000	0.979～1.021	0.969
PCI performed	0.471	0.312～0.710	<0.001
Hypertension	1.208	0.739～1.975	0.450
Stroke/TIA	0.900	0.571～1.419	0.652
Peripheral vascular disease	1.445	0.921～2.265	0.109

Model 2 included age, sex, BMI, smoking history, LVEF, PCI receipt, hypertension, stroke/TIA, and peripheral vascular disease. HR indicates hazard ratio; 95% CI indicates the 95% confidence interval for the hazard ratio.

### 3.7 Exploratory subgroup analysis of the descriptive risk-stratification value of CCI score for long-term All-cause mortality

To describe the consistency of the risk-stratification association between CCI score and all-cause mortality across different clinical strata, exploratory subgroup analyses were performed according to major comorbidities, age strata, and PCI status, with CCI score entered as a continuous variable. The direction of the association between higher CCI score and mortality was generally consistent across the prespecified subgroups, and no significant interaction was observed. Patients with chronic kidney disease, heart failure, or no PCI receipt had higher absolute mortality rates, suggesting that these factors contributed to higher baseline risk. However, because CCI did not show a statistically significant independent association with mortality in the overall fully adjusted model, these subgroup findings should be interpreted only as exploratory descriptive evidence of directional consistency. They should not be used as confirmatory evidence that CCI independently predicts long-term mortality. In addition, patients with chronic kidney disease, heart failure, or no PCI receipt had higher all-cause mortality rates, suggesting that these factors influenced baseline risk. Nevertheless, the subgroup results suggest that CCI may provide risk-stratification information across different clinical strata. The detailed results of the exploratory subgroup analyses are presented in Table 8.

**Table 8:** Exploratory subgroup analysis of the descriptive risk-stratification value of CCI score for long-term all-cause mortality.

Subgroup	Deaths/Total	Mortality (%)	Adjusted HR (95% CI)	*P* value	Interaction P
With CKD	48/109	44.04	1.30 (1.05–1.60)	0.018	0.72
Without CKD	50/241	20.75	1.38 (1.18–1.62)	<0.001	
With heart failure	46/96	47.92	1.28 (1.03–1.59)	0.026	0.58
Without heart failure	52/254	20.47	1.39 (1.19–1.63)	<0.001	
With diabetes mellitus	52/163	31.90	1.32 (1.10–1.58)	0.003	0.85
Without diabetes mellitus	46/187	24.60	1.36 (1.14–1.62)	<0.001	
Age <75 years	38/188	20.21	1.35 (1.12–1.62)	0.001	0.68
Age ≥75 years	60/162	37.04	1.29 (1.10–1.52)	0.002	
PCI performed	52/236	22.03	1.36 (1.15–1.61)	<0.001	0.80
No PCI performed	46/114	40.35	1.31 (1.05–1.63)	0.016	

HR indicates the hazard ratio for all-cause mortality per 1-point increase in CCI score. Subgroup analyses were exploratory and were intended to describe directional consistency across clinical strata rather than to establish independent predictive ability. Models were adjusted for age, except for age-stratified analyses, LVEF, and peak troponin. PCI receipt was not adjusted for in PCI-stratified analyses, whereas it was included in the other subgroup models. HRs, 95% CIs, and *P* values were derived from Cox regression models within each subgroup.

### 3.8 Kaplan–meier analysis of cumulative survival rates across groups

Kaplan–Meier cumulative survival rates at each follow-up time point are shown in Table 9. Cumulative survival declined progressively over time in all three groups, and the high comorbidity group had consistently lower cumulative survival rates than the low and moderate comorbidity groups at each time point (overall Log-rank *χ*^2^ = 16.824, *P* < 0.001). At 36 months of follow-up, the cumulative survival rates in the low, moderate, and high comorbidity groups were 85.59%, 72.54%, and 61.18%, respectively, with progressively widening between-group differences. These findings suggest that CCI categories can descriptively distinguish patients with different long-term survival trajectories; however, Kaplan–Meier analysis is unadjusted and does not substitute for multivariable assessment of independent predictive ability.

**Table 9:** Kaplan–meier cumulative survival rates at selected time points across groups (%).

Time point	Low (%)	Moderate (%)	High (%)
6 months	97.42	93.65	88.82
12 months	94.06	88.74	78.64
24 months	89.83	80.28	68.42
36 months	85.59	72.54	61.18
48 months	83.05	70.42	60.00

Cumulative survival rates were estimated using the Kaplan–Meier method. The log-rank test was used for between-group comparisons, and the *P* value represents the overall comparison among the three groups.

## 4 Discussion

### 4.1 Comorbidity burden in elderly AMI patients and the clinical value of CCI

Multimorbidity is a common clinical feature among elderly patients with cardiovascular disease. In the present cohort of 350 elderly patients with AMI, 25.71% were classified into the high comorbidity group (CCI ≥5), and more than 20% had three or more chronic conditions, reflecting the substantial comorbidity burden in this population. As a simple and stable tool for descriptive risk stratification, CCI was significantly associated with long-term all-cause mortality in univariable Cox analysis (HR = 1.164, *P* = 0.002), which is consistent with previous findings in oncology, chronic kidney disease, and ICU populations (10, 11).

It should be noted that in multivariable Cox Model 1, the independent prognostic value of CCI was attenuated after adjustment for age and other confounders (HR = 1.089, *P* = 0.125). This finding suggests that age may be an important confounder in the association between CCI and mortality. Although the unadjusted CCI used in this study does not directly include age, comorbidity burden generally increases with age in elderly AMI patients; therefore, age may be correlated with CCI score, and simultaneous inclusion of both variables in the model may weaken the independent effect of CCI. In this study, CCI is therefore positioned as a descriptive risk stratification tool rather than a confirmed independent predictor of long-term mortality. Although CCI continued to show risk-stratification value in Kaplan–Meier and subgroup analyses, its ability to serve as an independent prognostic indicator requires further validation using formal measures of discrimination and calibration.

In addition, the index AMI event was included in the CCI calculation in this study. Because all patients had AMI, this uniformly increased each patient's CCI score by 1 point and did not change the relative ranking of patients according to comorbidity burden. However, this approach shifted the absolute CCI values upward and may affect the interpretation of predefined CCI categories. More importantly, because CCI is generally intended to reflect pre-existing comorbidity burden, inclusion of the acute index AMI event limits the interpretation of CCI as a measure of baseline comorbidity burden. Furthermore, the inclusion of the index AMI event in CCI may introduce a constitutive association between CCI and study outcomes; accordingly, findings from this study should not be directly compared with studies that calculated CCI without including the index AMI event, and should be interpreted primarily as descriptive risk stratification evidence. Therefore, future studies should consider calculating CCI based on pre-existing comorbidities excluding the index AMI event, or using the age-adjusted CCI, to better distinguish the effects of age, baseline comorbidity burden, and the acute index event.

Hypertension was not identified as an independent prognostic factor in the multivariable Cox models. This null finding may be related to the so-called “hypertension paradox,” whereby some elderly AMI patients with established hypertension may have received long-term antihypertensive therapy and thus achieved partial cardiovascular risk control (12, 13). It may also reflect interactions with other covariates in the multivariable model. Further studies are needed to clarify the underlying mechanisms.

### 4.2 Relationship between comorbidity burden and clinical outcomes

Higher comorbidity burden was associated with worse outcomes from the in-hospital phase to long-term follow-up. In-hospital MACE rates rose from 11.9% in the low comorbidity group to 53.3% in the high comorbidity group. Several mechanisms likely underlie this gradient. First, the cumulative effect of multiple chronic diseases significantly reduces cardiac reserve and the capacity to compensate for the acute hemodynamic stress of AMI. Second, in patients with CKD, the interplay of chronic inflammation, anemia, and cardiorenal cross-talk can potentiate myocardial injury and impair recovery (14, 15). In addition, patients in the high comorbidity group had a longer hospital stay than those in the low comorbidity group, which may increase medical resource consumption and expose patients to a higher risk of hospital-acquired infection, thereby further worsening prognosis (16).

The Kaplan–Meier analysis demonstrated significant between-group survival differences during follow-up, with progressively lower cumulative survival rates in patients with higher CCI categories. The overall log-rank test showed a significant difference among the three groups (*χ*^2^ = 16.824, *P* < 0.001). At 48 months, the cumulative survival rates were 83.05%, 70.42%, and 60.00% in the low, moderate, and high comorbidity groups, respectively. These findings suggest that higher comorbidity burden is associated with worse long-term survival in elderly AMI patients (17, 18).

### 4.3 The prognostic impact of CKD and heart failure

Among all comorbidity subgroups, patients with CKD or heart failure had the highest mortality burden, with all-cause mortality rates of 44.04% and 47.92%, respectively. Both conditions have well-established bidirectional relationships with AMI. CKD may increase mortality risk by accelerating coronary atherosclerosis and promoting myocardial fibrosis, while renal dysfunction may also limit the use of standard-dose antiplatelet agents, statins, and renin–angiotensin system inhibitors because of safety concerns, thereby affecting access to guideline-directed therapy (19, 20). Heart failure similarly worsens long-term prognosis through accelerated ventricular remodeling and progressive decline in cardiac output (21).

Notably, CCI score remained significantly associated with all-cause mortality in the prespecified subgroup analyses, and no significant interaction was observed across clinical subgroups. However, because the independent association between CCI and mortality was attenuated in the overall multivariable model, these subgroup findings should be considered exploratory. These results suggest that CCI may still provide useful risk-stratification information across different clinical strata, but its independent prognostic value requires further validation in larger multicenter cohorts. Therefore, early recognition and active management of high-weight comorbidities such as CKD and heart failure should be emphasized in clinical risk assessment for elderly AMI patients. When necessary, individualized treatment strategies should be developed within a multidisciplinary care framework to improve the long-term prognosis of these high-risk patients (22). In addition, formal subgroup interaction analyses showed no significant interaction across clinical subgroups (all interaction *P* values >0.05), further supporting the directional consistency of these associations across clinical strata, though these findings should not be interpreted as confirmatory evidence of CCI's independent prognostic ability.

### 4.4 Interpretation of other prognostic factors in multivariable analysis

Multivariable Cox regression analysis showed that smoking history and PCI receipt were independent prognostic factors. Regarding sex, male sex did not reach statistical significance in the multivariable model (HR = 0.841, *P* = 0.403), which differs from some previous studies and may be related to the age and comorbidity distribution of the present elderly cohort (23). Smoking history was independently associated with worse prognosis (HR = 1.862, *P* = 0.003), and its adverse impact on AMI outcomes may be mediated by platelet activation, endothelial dysfunction, and other mechanisms (24).

Regarding LVEF and cardiac function, univariable Cox analysis showed that each 1-unit increase in LVEF corresponded to an HR of 0.989 (95% CI: 0.969–1.009), indicating the expected direction of association. However, LVEF was not independently associated with mortality in the multivariable models (HR≈1.000, *P* > 0.7). This suggests that the prognostic value of LVEF in elderly AMI patients may be partially covered by other factors, such as comorbidity burden and PCI receipt. Clinically, LVEF assessment should still be combined with CCI scoring to achieve more accurate composite risk stratification (25).

PCI receipt was independently associated with a lower risk of long-term all-cause mortality (HR = 0.497, *P* = 0.001), however, as this was a retrospective observational study with non-randomized treatment allocation, patients who did not receive PCI may have differed systematically in age, renal function, coronary anatomy complexity, bleeding risk, or patient preference. This finding is therefore best interpreted as an independent association between PCI receipt and prognosis rather than as causal evidence of a protective effect of PCI itself. BMI showed a borderline trend in Model 2 (HR = 0.947, *P* = 0.078), suggesting that lower BMI may be associated with higher mortality risk, possibly reflecting the combined effects of malnutrition and sarcopenia (26).

### 4.5 Clinical implications and limitations

The main clinical implication of this study is that CCI score was associated with long-term all-cause mortality in elderly AMI patients in univariable Cox analysis and Kaplan–Meier stratified analysis, suggesting that it has value for descriptive risk stratification. At the same time, smoking history and absence of PCI were identified as independent prognostic risk factors; the association with PCI receipt should be interpreted in light of potential indication bias and the limitations of the observational design. Age, sex, and LVEF did not show independent effects in the multivariable analysis, which may be because their prognostic effects were partly covered by comorbidity burden and other covariates. In clinical practice, CCI may be used as an auxiliary tool to rapidly identify patients with high comorbidity burden. Patients with CCI ≥5 may be considered for more intensive follow-up monitoring and comprehensive management pathways, including concurrent management of multiple conditions, renal protection, heart failure management, smoking cessation, nutritional assessment, and cardiac rehabilitation. However, individual clinical decision-making should integrate age, AMI severity, coronary anatomy, PCI indications and contraindications, LVEF, renal function, and patient preferences; individualized prognostic assessment should not be based on CCI score alone.

Several limitations should be acknowledged. First, the single-center retrospective design introduces unavoidable selection and information biases, and the generalizability of the findings requires validation in multicenter studies. Second, some long-term follow-up data were obtained by telephone interview, which may limit the accuracy of cause-of-death ascertainment. Third, several potential confounders, including psychosocial factors, medication adherence, and nutritional assessment scores, were not included and may have influenced the prognostic analysis. Fourth, the relatively modest sample size may have limited statistical power in some low-frequency comorbidity subgroups. Fifth, this study did not calculate discrimination or calibration metrics for CCI; therefore, the incremental prognostic performance of CCI beyond conventional clinical variables requires further evaluation using formal measures such as the C-statistic, net reclassification improvement, and calibration plots. Sixth, the use of the unadjusted CCI may have introduced age-related confounding, and future studies should consider using the age-adjusted CCI to better separate the independent effects of age and comorbidity burden. Seventh, the index AMI event was included in the CCI calculation, which uniformly shifted all CCI scores upward and may affect the interpretation of absolute CCI categories. Future studies should consider calculating CCI based on pre-existing comorbidities excluding the index AMI event. Eighth, patients who died within 48 h of hospitalization were excluded, which may have introduced survivorship bias and limited the applicability of the findings to the most critically ill elderly AMI patients. Ninth, no sensitivity analysis was conducted after excluding the index AMI event from the CCI calculation; the inclusion of the index AMI event in the CCI score may therefore have introduced a constitutive association between CCI and study outcomes, and findings from the primary analysis should be interpreted principally as descriptive risk stratification evidence. Future studies should conduct multicenter prospective cohort analyses, incorporate more comprehensive prognostic variables, compare unadjusted CCI with age-adjusted CCI, and report discrimination and calibration metrics for CCI to establish a more accurate prognostic model for elderly AMI patients.

## 5 Conclusions

In this cohort, higher CCI scores were associated with greater long-term all-cause mortality and adverse clinical outcomes in elderly patients with AMI, supporting the use of CCI for descriptive risk stratification and identification of patients with high comorbidity burden. However, as CCI did not demonstrate an independent statistical association after multivariable adjustment, these findings should not be interpreted as confirmatory evidence that CCI independently predicts long-term mortality. Smoking history and absence of PCI receipt were independently associated with worse prognosis; the association with PCI receipt should be interpreted cautiously given potential indication bias, contraindications, and selection effects inherent to the observational design. Routine CCI assessment may assist in identifying high-risk patients, but individualized clinical decision-making should integrate multiple clinical factors rather than CCI score alone.

## Data Availability

The original contributions presented in the study are included in the article/Supplementary Material, further inquiries can be directed to the corresponding author.
